# Fungal Pigments: Deep into the Rainbow of Colorful Fungi

**DOI:** 10.3390/jof3030045

**Published:** 2017-08-07

**Authors:** Laurent Dufossé, Yanis Caro, Mireille Fouillaud

**Affiliations:** 1 Laboratoire de Chimie des Substances Naturelles et des Sciences des Aliments—LCSNSA EA 2212, Université de la Réunion, 15 Avenue René Cassin, CS 92003, F-97744 Saint-Denis CEDEX 9, Ile de la Réunion, France; 2 Ecole Supérieure d’Ingénieurs Réunion Océan Indien—ESIROI agroalimentaire, 2 Rue Joseph Wetzell, F-97490 Sainte-Clotilde, Ile de la Réunion, France

With the impact of globalization on research trends, the search for healthier life styles, the increasing public demand for natural, organic, and ”clean labelled” products, as well as the growing global market for natural colorants in economically fast-growing countries all over the world, filamentous fungi started to be investigated as readily available sources of chemically diverse pigments and colorants. For all of these reasons, this Special Issue of the *Journal of Fungi* highlights exciting new findings, which may pave the way for alternative and/or additional biotechnological processes for industrial applications of fungal pigments and colorants. Eight research papers and one review constitute the journal’s final Special Issue.

Our first target when building this project was to welcome papers on the following topics: 

The fungal biodiversity from terrestrial and marine origins, bringing new elements about fungi as potential sources of well-known carotenoid pigments (e.g., β-carotene, lycopene) and other specific pigmented polyketide molecules, such as *Monascus* and *Monascus*-like azaphilones, which are yet not known to be biosynthesized by any other organisms such as higher plants. These polyketide pigments also include promising and unexplored hydroxy-anthraquinoid colorants from Ascomycetous species.

The investigation of biosynthetic pathways of the carotenoids and polyketide-derivative colored molecules (i.e., azaphilones, hydroxyanthraquinones, and naphthoquinones) in pigment-producing fungal species.

The description of alternative greener extraction processes of the fungal colored compounds, along with current industrial applications, description of their limits and further opportunities for the use of fungal pigments in beverage, food, pharmaceutical, cosmetic, textile and painting areas.

All these subjects and more are covered by articles published in this Issue: 

http://www.mdpi.com/journal/jof/special_issues/fungal_pigments.

* Fungal biodiversity from terrestrial and marine origins:

**Production and New Extraction Method of Polyketide Red Pigments Produced by Ascomycetous Fungi from Terrestrial and Marine Habitats** by Lebeau J. et al. doi:10.3390/jof3030034.

**Biodiversity of Pigmented Fungi Isolated from Marine Environment in La Réunion Island, Indian Ocean: New Resources for Colored Metabolites** by Fouillaud et al. doi:10.3390/jof3030036.

* Biosynthesis of fungal pigments and ways to increase the efficacy of biosynthetic routes and/or the diversity of the biosynthesized pigments:

**Combinatorial Biosynthesis of Novel Multi-Hydroxy Carotenoids in the Red Yeast *Xanthophyllomyces dendrorhous*** by Pollmann et al. doi:10.3390/jof3010009.

**Carotenoid Biosynthesis in *Fusarium*** by Avalos J. et al. doi:10.3390/jof3030039.

**Biosynthesis of Astaxanthin as a Main Carotenoid in the Heterobasidiomycetous Yeast *Xanthophyllomyces dendrorhous*** by Barredo J.L. et al. doi:10.3390/jof3030044.

* In situ *microscopic analysis of fungal pigments applied on surfaces:*

**Microscopic Analysis of Pigments Extracted from Spalting Fungi** by Vega Gutierrez S.M. and Robinson, S.C. doi:10.3390/jof3010015.

* New modes of extraction of fungal pigments (perstraction, pressurized liquid extraction technique):

**Perstraction of Intracellular Pigments through Submerged Fermentation of *Talaromyces* spp. in a Surfactant Rich Media: A Novel Approach for Enhanced Pigment Recovery** by Morales-Oyervides L. et al. doi:10.3390/jof3030033.

Part of **Production and New Extraction Method of Polyketide Red Pigments Produced by Ascomycetous Fungi from Terrestrial and Marine Habitats** by Lebeau J. et al., with investigation of a pressurized liquid extraction technique. doi:10.3390/jof3030034.

* Fine chemical analysis of extracted fungal pigments:

**Utilization of High Performance Liquid Chromatography Coupled to Tandem Mass Spectrometry for Characterization of 8-O-methylbostrycoidin Production** by Species of the Fungus *Fusarium* by Busman, M. doi:10.3390/jof3030043.

* Application of fungal pigments in the industry:

**Assessment of the Dyeing Properties of the Pigments Produced** by *Talaromyces* spp. by Morales-Oyervides L. et al. doi:10.3390/jof3030038.

We, as Guest Editors, trust all readers of this Special Issue enjoy the contents and we would like to deeply thank all 34 authors who contributed (sorted by their last name), also Prof. Dr. David S. Perlin, Editor-in-Chief of the *Journal of Fungi,* and the editing team at MDPI: 

Avalos, JavierKosalkova, KatarinaPollmann, HendrikBarredo, Jose L.Lebeau, JulianaRobinson, Sara C.Barreiro, CarlosLimón, María CarmenRodríguez-Ortiz, RobertoBode, Helge B.Llorente, MelissaRuger-Herreros, MacarenaBreitenbach, JürgenMagalon, HeleneSandmann, GerhardBusman, MarkMéndez-Zavala, AlejandroSousa-Gallagher, MariaCaro, YanisMontañez, Julio CesarVega Gutierrez, Sarath M.Cuet, PascaleMorales-Oyervides, LourdesVenkatachalam, MekalaDufossé, LaurentOliveira, JorgeVinale, FrancescoFouillaud, MireillePardo-Medina, JavierWolff, HendrikGarcía-Estrada, CarlosParra-Rivero, ObduliaHornero-Méndez, DámasoPetit, Thomas

